# IMPROVING WELL-BEING IN OLDER ADULTS: USING AN ITERATIVE PROCESS TO OPTIMIZE THE DESIGN OF A GROUP INTERVENTION

**DOI:** 10.1093/geroni/igae098.0133

**Published:** 2024-12-31

**Authors:** Erin Cassidy-Eagle, Christine Gould, Feng Vankee Lin, Maryam Makowski, Dolores Gallagher-Thompson

**Affiliations:** Stanford University School of Medicine, Stanford, California, United States; VA Palo Alto Healthcare System, Palo Alto, California, United States; Stanford University School of Medicine, Stanford, California, United States; Stanford University School of Medicine, Stanford, California, United States; Stanford University School of Medicine, Stanford, California, United States

## Abstract

Psychological health and well-being definitions are moving beyond the goal of simply decreasing symptoms and disorder rates and moving towards a life enhancing and positive focus. Our multi-modal group program targets a broad range of well-being dimensions including cognitive, behavioral, emotional, social, and physical health parameters. The changes to design and implementation made to the intervention as part of an iterative process will be discussed. Participants were older adults (65+), absent a dementia diagnosis, referred from providers across the healthcare system. The intervention focuses on a collection of topics that support optimal psychological wellness and promote health and resilience (i.e. digital tools and mobile apps, value-based behavioral activation to improve your mood, eating for a healthy brain, introduction to mindfulness, improving your sleep and strategies for managing anxiety) that are held weekly, with modules ranging in duration from 2-4 weeks, over a 7-month period. The results across 2 groups (N=40), both process and outcome, will be presented. Positive psychiatry interventions have great potential to support and strengthen the psychological well-being of older adults who are faced with the myriad of challenges that come with increasing age. Considering the geriatric mental health provider shortages and the overwhelmed healthcare system, this program has improved access to psychiatric care. Program goals also include aims of boosting positive emotions, life satisfaction, purpose and meaning, along with decreasing loneliness, depression, and anxiety.

